# Separable Nonlinear Least-Squares Parameter Estimation for Complex Dynamic Systems

**DOI:** 10.1155/2020/6403641

**Published:** 2020-04-02

**Authors:** Itai Dattner, Harold Ship, Eberhard O. Voit

**Affiliations:** 1 Department of Statistics, University of Haifa, 199 Aba Khoushy Ave., Mount Carmel, Haifa 3498838, Israel; 2 The Wallace H. Coulter Department of Biomedical Engineering, Georgia Institute of Technology and Emory University, 950 Atlantic Drive, Atslanta, GA 30332–2000, USA

## Abstract

Nonlinear dynamic models are widely used for characterizing processes that govern complex biological pathway systems. Over the past decade, validation and further development of these models became possible due to data collected via high-throughput experiments using methods from molecular biology. While these data are very beneficial, they are typically incomplete and noisy, which renders the inference of parameter values for complex dynamic models challenging. Fortunately, many biological systems have embedded linear mathematical features, which may be exploited, thereby improving fits and leading to better convergence of optimization algorithms. In this paper, we explore options of inference for dynamic models using a novel method of *separable nonlinear least-squares optimization* and compare its performance to the traditional nonlinear least-squares method. The numerical results from extensive simulations suggest that the proposed approach is at least as accurate as the traditional nonlinear least-squares, but usually superior, while also enjoying a substantial reduction in computational time.

## 1. Introduction

Nonlinear dynamic models are widely used for characterizing the processes that govern complex biological pathway systems. Of particular interest in this context are so-called canonical formats, which are very flexible in their possible responses, yet involve a very restricted domain of functional forms. Outside linear systems, the best-known canonical formats are Lotka–Volterra (LV) models [1–4], which use binomial terms, and power-law systems within the framework of Biochemical Systems Theory (BST), which exclusively use products of power functions. BST was originally devised for the analysis of biochemical and gene regulatory systems, but has subsequently found much wider application in various biomedical and other areas [5, 6]. Whereas it is easy to set up an LV or BST model for a complex biological system in a symbolic format, the identification of optimal parameter values continues to be a significant challenge. As a consequence, estimating parameters of systems of ordinary differential equations (ODEs) remains to be an active research area that attracts contributions from a variety of scientific fields (e.g., [7–12]. Indeed, numerous optimization methods for ODE models have been proposed in recent years, but none works exceptionally well throughout a wide range of applications, with reasons spanning the entire spectrum from intrinsic problems with biological data (sparseness, uncertainties, noise, …) to technical and computational issues (numerous local minima, unidentifiability, sloppiness, …). Methods like slope-based estimation (e.g., [13]) and dynamic flux estimation [14–16] alleviate these problems but are not panacea.

Here, we revisit, and bring to fruition, early ideas [17] of separating estimation tasks into linear and nonlinear aspects. However, our main focus is *not* really a new estimation method *per se*. Instead, we are interested in a more general and higher-level point of view regarding parameter estimation than that typically presented in technical articles. Specifically, this article addresses parameter estimation for dynamic models whose mathematical format contains linear features that allow a natural separation of parameters and system states. A trivial example is a linear ODE where the vector field *x*′(*t*) = *θx*(*t*) is linear in the parameter *θ*, with *x*′(*t*) denoting the derivative of *x*(*t*) with respect to *t*. As a more interesting example, the ODE vector field may be partially linear in the parameters, as it is the case for so-called S-system models in BST [5].

*Example 1.* An S-system [18] is defined as

(1) $$x_{j}^{\prime }(t)=\alpha_{j}\prod_{k=1}^{d}x_{k}^{g_{jk}}(t)-\beta_{j}\prod_{k=1}^{d}x_{k}^{h_{jk}}(t),\;j=1,\ldots ,d.$$

Here *α*_*j*_*, β*_*j*_ are non-negative rate constants, while *g*_*jk*_*, h*_*jk*_ are real-valued kinetic orders that reflect the strength and directionality of the effect that a variable has on a given influx or efflux. Informally, one can view this system as a regression equation, where the “covariates” are the variables *x*_*j*_(*t*) on the right-hand side, whereas the “response” variables are the derivatives $x_{j}^{\prime }(t)$ on the left-hand side. Note that the vector field is linear in the rate constants *α*_*j*_*, β*_*j*_, but nonlinear in the kinetic orders *g*_*jk*_*, h*_*jk*_.

Estimation methods that exploit separability of parameters and system states in dynamic models have a long history; see [19] for a special case. However, a rigorous statistical analysis of such a method has been achieved only recently [20]. In a classical paper on the inference for dynamic models, Varah [17] mentioned in passing that “one can use the idea of separability or variable projection (see [21] or [22]), in which the linear parameters are implicitly solved for, the resulting (fully) nonlinear least-squares problem is solved for the nonlinear parameters, and then the linear parameters are obtained using their representation in terms of the nonlinear parameters. Since this reduces the size of the nonlinear least-squares problem to be solved, it is worthwhile.” Somewhat surprisingly, given that parameter estimation for ODEs is commonly thought as a computational bottleneck in modeling dynamic processes, Varah’s suggestion has not been widely followed in practice. In fact, in the vast literature dedicated to parameter fitting techniques for dynamic models, we are aware of only two relevant references: using a direct integral approach, Dattner et al. [23] applied a separable nonlinear least-squares technique to the inference of parameters in a predator-prey system acting in a heterogeneous environment, while Wu et al. [24] used separability to estimate parameters of high-dimensional linear ODE systems. Moreover, Varah’s idea of exploiting separability for estimating ODE parameters has been implemented only recently in a publicly available software package [25]. Pertinent details of this software will be discussed in a later section.

The analysis in this paper is hoped to convince the reader that Varah’s idea is indeed worth pursuing. To support this claim, we explore and compare two general data fitting approaches for dynamic models: the traditional nonlinear least-squares method (NLS) and the proposed separable nonlinear least-squares method (SLS). Through extensive Monte-Carlo simulations of representative complex models, we identify and quantify significant statistical and computational gains obtained with this separation method. We will ultimately come to the conclusion that model separability can be very beneficial and that the SLS approach should be considered for any complex dynamic system that possess significant linear features.

The paper is organized as follows. In Section 2, we present details of the SLS methodology in the context of dynamic models. Section 3 describes the simulation setup, quantifies the statistical measures we use in order to compare the performance of SLS and NLS, and presents numerical results. In Section 4, we point out future research directions, while conclusions are provided in Section 5.

## 2. Separable Nonlinear Least-Squares (SLS) and Varah’s Idea

### 2.1. Generalities

Following Varah’s original idea within the context of inference in dynamic models, the main advantages of exploiting separability for parameter estimation are the following [26]:

- Fewer initial guesses are required for optimization
- The optimization problem is better conditioned
- Convergence is faster

These advantages have been convincingly demonstrated in several publications. For example, see Mullen [27] for an implementation and applications in physics and chemistry; Chung & Nagy [28] for a high-dimensional case, where the number of parameters is substantially larger than the number of observations; Gan et al. [29] who compared the performance of several algorithms for SLS problems; and Erichson et al. [30] who studied sparse principal component analysis via variable projection. Separable models are of broad practical applicability, and as such form a subject of active theoretical and applied research. For instance, when analyzing the “reduced” nonlinear optimization problem of a separable structure, simplified conditions are required for establishing a variety of theoretical results concerning numerical and statistical properties of the resulting estimators, compared to the original NLS problem (e.g., [20, 31]).

In the following, we focus on complex dynamic models that are formulated as systems of ordinary differential equations (e.g., [32]). Specifically, consider a system of equations given by

(2) $$\{\begin{array}{l} x^{\prime }(t)=F(x(t);\theta ),\;t\in [0,T],\\ x(0)=\xi , \end{array}$$

where *x*(*t*) takes values in ${\mathbb{R}}^{d}$, $\xi \in \Xi \subset {\mathbb{R}}^{d}$, and $\theta \in \Theta \subset {\mathbb{R}}^{p}$. For our purposes, we explicitly separate linear components from nonlinear ones in the function *F* by setting

(3) $$F(x(t);\theta )=g\left(x(t);\theta_{\text{NL}}\right)\theta_{\text{L}},$$

where $\theta ={\left(\theta_{\text{NL}}^{⊤},\theta_{\text{L}}^{⊤}\right)}^{⊤}$, and the symbol ⊤ stands for the matrix transpose (cf., [20]). Here *θ*_NL_, a vector of size *p*_NL_, stands for the “nonlinear” parameters that are not separable from the state variables *x*, while *θ*_L_, a vector of size *p*_L_, contains the “linear” parameters; note that *p* = *p*_L_ + *p*_NL_. As the vector field in (3) is separable in the linear parameter vector *θ*_L_, we refer to the corresponding ODE system as *linear in the parameter θ*_L_ (cf. the case of a linear regression model), although the *solution* to the system might be highly nonlinear in θ, or even implicit.

*Example 2.* Let

$$\theta_{\text{NL}}=(g_{11},\ldots ,g_{1d},\ldots ,g_{d1},\ldots ,g_{dd},h_{11},\ldots ,\;\;{h_{1d},\ldots ,h_{d1},\ldots ,h_{dd})}^{⊤},$$

$$\theta_{\text{L}}={\left(\alpha_{1},\beta_{1},\ldots ,\alpha_{d},\beta_{d}\right)}^{⊤}.$$

Then, one sees that equation (1) is a special case of (2)–(3).

### 2.2. Solution Strategy

Let *x*(*t*; *θ, ξ*), *t* ∈ [0*, T*], be the solution of the initial value problem (2). We assume that noisy measurements *Y*_*j*_(*t*_*i*_) on the system are collected at time points *t*_*I*_ ∈ [0*, T*]. A common statistical formulation of this situation is

(4) $$Y_{j}\left(t_{i}\right)=x_{j}\left(t_{i};\theta ,\xi \right)+\varepsilon_{ij},\;i=1,\ldots ,n,\;j=1,\ldots ,d.$$

Here the random variables *ε*_*ij*_ are unobservable, independent measurement errors (not necessarily Gaussian) with zero mean and finite variance.

Varah’s approach to parameter estimation in ODE models works as follows. Let $\hat{x}(t)$ stand for a smoother of the data, obtained, e.g., using splines or local polynomials (see e.g., [33, 34] and [35] for a treatment of various smoothing methods and an extensive bibliography). This smoother approximates the solution *x*(*t*; *θ, ξ*) to the ODE (2). Varah suggests to insert the smoother into equation (2), which will now be satisfied only approximately, and to minimize the resulting discrepancy over the parameters *ξ* and *θ*. A minimizer $\left(\hat{\xi },\hat{\theta }\right)$ is then an estimator of (*ξ, θ*). This idea was put on a solid statistical foundation in Brunel [36] and Gugushvili and Klaassen [37]. Varah’s original approach requires the use of the derivative ${\hat{x}}^{\prime }(t)$ as an estimator of *x*′(*t*), which is a disadvantage, as it is well known that estimating derivatives from noisy and sparse data may be rather inaccurate; see e.g., Vilela et al. [38] and Chou and Voit [7] or more generally Fan and Gijbels [33]. Recent research [20, 23, 39–46] has shown that it is more fruitful to transplant Varah’s idea to the solution level of equation (2). To accomplish this shift, we define an integral criterion function

(5) $$\int_{0}^{T}{\left\|\hat{x}(t)-\xi -\int_{0}^{t}F(\hat{x}(s);\theta )\text{d}s\right\|}^{2}\text{d}t,$$

as it is typical in estimation approaches based on integrals (see references above). Here, ||·|| is the Euclidean norm. A minimizer of (5) over (*ξ, θ*) yields a parameter estimator that typically has slightly different features than an estimator based on the differential equations themselves. In practice, the integral is discretized and replaced by a sum, so that minimization can be performed using a typical nonlinear least-squares method, such as *fminsearch* in Matlab. The discretized format is

(6) $$\left({\hat{\xi }}_{\text{NLS}},{\hat{\theta }}_{\text{NLS}}\right)=\arg\underset{\xi ,\theta }{\min}\int_{o}^{T}{\left\|\hat{x}(t)-\xi -\int_{0}^{t}F(\hat{x}(s);\theta )\text{d}s\right\|}^{2}\text{d}t.$$

The NLS solution does not take into account the specific linear form of the ODEs in (3), but uses the general form in (2).

It is at this stage that Varah suggested to utilize separability, without actually investigating such an approach. Here, we provide the necessary details (cf. [20]). Denote

$$\hat{G}(t):=\hat{G}\left(t;\theta_{\text{NL}}\right)=\int_{0}^{t}g\left(\hat{x}(s);\theta_{\text{NL}}\right)\text{d}s,\;t\in [0,T],\;\;\hat{A}=\int_{0}^{T}\hat{G}(t)\text{d}t,\;\;\hat{B}=\int_{0}^{T}{\hat{G}}^{⊤}(t)\hat{G}(t)\text{d}t.$$

Then, with *θ*_NL_ kept fixed, a minimizer of (5) is given by

$$\;\hat{\xi }\left(\theta_{\text{NL}}\right)={\left(TI_{d}-\hat{A}{\hat{B}}^{-1}{\hat{A}}^{⊤}\right)}^{-1}\;\;\int_{0}^{T}\left(I_{d}-\hat{A}{\hat{B}}^{-1}{\hat{G}}^{⊤}(t)\right)\hat{x}(t)\text{d}t,\;{\hat{\theta }}_{\text{L}}\left(\theta_{\text{NL}}\right)={\hat{B}}^{-1}\int_{0}^{T}{\hat{G}}^{⊤}(t)(\hat{x}(t)-\hat{\xi })\text{d}t,$$

where *I*_*d*_ denotes the *d* × *d* identity matrix. The notation $\hat{\xi }\left(\theta_{\text{NL}}\right)$ and ${\hat{\theta }}_{\text{L}}\left(\theta_{\text{NL}}\right)$ emphasizes the dependence of the solution on the nonlinear parameters *θ*_NL_. This solution $\left(\hat{\xi }\left(\theta_{\text{NL}}\right),{\hat{\theta }}_{\text{L}}\left(\theta_{\text{NL}}\right)\right)$ is plugged back into (5), yielding the reduced integral criterion function (cf. [23]):

(7) $$M\left(\theta_{\text{NL}}\right):=\int_{0}^{T}{\left\|\hat{x}(t)-\hat{\xi }\left(\theta_{\text{NL}}\right)-\hat{G}\left(t;\theta_{\text{NL}}\right){\hat{\theta }}_{\text{L}}\left(\theta_{\text{NL}}\right)\right\|}^{2}\text{d}t.$$

Once *M*(*θ*_NL_) is minimized over *θ*_NL_ and a solution

$${\hat{\theta }}_{\text{NL}}=\arg\underset{\theta_{\text{NL}}}{\min}\;M\left(\theta_{\text{NL}}\right),$$

is obtained, estimators for *ξ* and *θ* follow immediately and are given (with mild abuse of the matrix transpose notation) by

(8) $${\hat{\xi }}_{\text{SLS}}=\hat{\xi }\left({\hat{\theta }}_{\text{NL}}\right),\;{\hat{\theta }}_{\text{SLS}}=\left({\hat{\theta }}_{\text{NL}},{\hat{\theta }}_{\text{L}}\left({\hat{\theta }}_{\text{NL}}\right)\right),$$

respectively. Equations (7) and (8) are driven by Varah’s [17] suggestion discussed above. Indeed, note that the nonlinear optimization is applied only for estimating the nonlinear parameters *θ*_NL_, which, in comparison to the NLS approach, can substantially reduce the dimension of the nonlinear optimization problem.

From the above derivation, it is clear that SLS problems constitute a special class of NLS problems, with linear and nonlinear objective functions for different sets of variables. While the idea of using separability for improving parameter estimation was presented already in Lawton and Sylvestre [47], it seems that much of the subsequent literature is based on the variable projection method proposed by Golub and Pereyra [21]. Golub and Pereyra [26] reviewed 30 years of research into this problem.

## 3. Simulation Framework and Results

In order to investigate the relative performance of SLS and NLS, we designed and performed a large Monte-Carlo simulation, whose results are presented in this section.

All computations were carried out in *R* on an Amazon EC2 m5a.4xlarge instance using the *simode* package of Yaari and Dattner [25] (Separable Integral Matching for Ordinary Differential Equations). The statistical methodologies applied in the package use smoothing and minimization of an integral-matching criterion function, taking advantage of the mathematical structure of the differential equations like separability of parameters from equations. Application of smoothing and integral-based methods to parameter estimation of ordinary differential equations was shown to yield more accurate and stable results comparing to derivative based ones [20]. Here, we used default smoothing and optimization settings in *simode*, and in that respect, both SLS and NLS received equal treatment. Specifically, *simode* uses cross validation (see, e.g., [35]) to determine the optimal amount of smoothing. A detailed guide for using the package can be found in Yaari and Dattner [25]. The code to reproduce our numerical results can be accessed on GitHub (see https://github.com/haroldship/complexity-2019-code/tree/master/Final Code First Submission). For plotting, we relied on the *ggplot2* package in *R*, see Wickham [48].

### 3.1. Monte-Carlo Study Design

We chose several representative and challenging ODE models arising in a variety of scientific disciplines. Those were

- An SIR model for simulating the spread of an infectious disease
- A Lotka–Volterra population model with sinusoidal seasonal adjustment
- A Generalised Mass Action (GMA) system within BST, e.g., for metabolic pathway systems
- A FitzHugh–Nagumo system of action potentials along neuronal axons

Further mathematical details on these systems and the specific experimental setups we used are given below.

In each case, we generated observations by numerically integrating the system and then adding independent Gaussian noise to the time courses, as in (4). We considered various parameter setups, sample sizes, and noise levels, as specified below. The ODE parameters were estimated via both NLS and SLS, as defined in equations (6) and (8), respectively.

As performance criteria, the time required to perform optimization and the accuracy of the resulting parameter estimates were used. While comparing computation times is trivial, numerous options are available for comparing accuracy. We focused on the main difference between the two optimization schemes, namely the way they deal with the estimation of linear parameters. SLS does not require initial guesses for these parameters. By contrast, NLS does require a good initial guess for each linear parameter; otherwise, it might diverge or get stuck in a local minimum. Thus, finding “good” solutions to nonlinear optimization problems often requires “good” initial guesses in the parameter space. Clearly, some “prior information” regarding these parameters is of crucial importance for optimization purposes. The key insight is that this prior information is encapsulated in the mathematical form of the ODEs themselves, such as (3). Importantly, while NLS does not take into account the special mathematical features of the ODEs and treats all the parameters in a uniform manner, this is not the case for SLS. Thus, one might *a priori* expect SLS to be more efficient and possibly more accurate than NLS, when prior information regarding the linear parameters is of low quality. On the other hand, when one has high-quality prior information regarding the linear parameters, we expect that SLS and NLS will perform similarly. One might note that the nonlinear parameters in almost all GMA and S-systems are very tightly bounded, usually between −1 and +2, and that their sign is often known, whereas the linear parameters are unbounded in GMA systems and nonnegative in S-systems, and nothing is known about their magnitudes (see Chapter 5 of [18]). Thus, not needing prior information on the linear parameters in SLS can be a tremendous advantage.

For the Monte-Carlo study, we varied the prior information by using high-, medium-, and low-quality initial guesses for the parameter values. Here, higher quality means that the initial guesses were closer to the truth. To be more specific, the initial guesses for the linear parameters used by NLS were Gaussian random variables centered on the true parameter values and having standard deviations equal to the true parameter multiplied by a prior information value (in other words, the prior information value can also be understood as the coefficient of variation of the “prior distribution”). The specific quantification of “high,” “medium,” and “low” is admittedly somewhat subjective and varies across the different ODE models, as specified below. For the sake of better and faster convergence of the optimization algorithms (especially NLS), the nonlinear parameters were constrained to a given range, and this range was the same no matter how we varied the prior information on linear parameters. Further, in each Monte-Carlo iteration, we used exactly the same (pseudorandom) initial guess for nonlinear parameters for both NLS and SLS. Thus, as far as the information on nonlinear parameters is concerned, this was kept invariant for each benchmark model, irrespective of the prior on linear parameters. Consequently, both algorithms received the same prior information regarding nonlinear parameters, and neither one was treated preferentially.

The noise level (signal-to-noise ratio, SNR) we used is defined as follows. For a given solution *x*(*t*) of an ODE equation, we calculate the standard deviation *σ*_*x*_ = std(*x*(*t*_1_), … *, x*(*t*_*n*_)). Then SNR of, say, 10% and 20% is given by *σ* = *σ*_*x*_/10, and *σ* = *σ*_*x*_/5, respectively, where *σ* is the standard deviation of a Gaussian measurement error ϵ as defined in equation (4). We will refer to these SNRs as “low noise” and “high noise,” respectively (cf. [49]; albeit in a different context). We then compared the mean square errors (MSE) of the resulting parameter estimates, which leads to a valid comparison in statistically identifiable ODE models (see e.g., [20] for relevant definitions and results). As another accuracy measure, we used the criteria (5) and (7) evaluated at optimal parameter values. The two criteria we propose, though reasonable, are different. Hence, they are not expected to be in agreement in every experimental setup. However, the global conclusions reached with them in Section 5 are coherent and favor SLS.

We now provide the mathematical details on the models and the experimental setups.

#### 3.1.1. Age-Group SIR

The system of interest is an epidemiological model of SIR-type (Susceptible—Infected—Recovered) and includes age groups and seasonal components (e.g., [50]). The infectious process in each age group 1 ≤ *a* ≤ *M* and each season 1 ≤ *y* ≤ *L* is described using two equations for the proportion of susceptible (*S*) and infected (*I*) individuals within the population (the proportion of recovered individuals is given by 1 − *S* − *I*):

(9) $$S_{a,y}^{\prime }(t)=-S_{a,y}(t)\kappa_{y}\sum_{j=1}^{M}\beta_{a,j}I_{j,y}(t),\;I_{a,y}^{\prime }(t)=S_{a,y}(t)\kappa_{y}\sum_{j=1}^{M}\left(\beta_{a,j}I_{j,y}(t)\right)-\gamma I_{a,y}(t).$$

The parameters of the model are the *M* × *M* transmission matrix *β*, the recovery rate *γ*, and *κ*_2,…*,L*_, which signify the relative infectivity of, e.g., influenza virus strains circulating in seasons 2, … *, L* compared to season 1 (*κ*_1_ is used as a reference and fixed at 1). As shown in Yaari et al. [46], taking into account separability characteristics of this model is advantageous for data fitting purposes. Specifically, (9) is nonlinear in the initial value *S*(0), which are typically unknown and have to be estimated. For our purposes, it suffices to consider a model with one age group and one season. The following parameter setup was used: *S*(0) = 0.56*, I*(0) = 1*e* − 04*, β* = 6*, γ* = 2.3. We considered two sample sizes, 18 and 36, and two noise levels, 10% and 20%. The prior information used was {0.1, 0.2, 0.3}, corresponding to high, medium, and low quality, respectively. The size of the Monte-Carlo study was 500 simulations.

#### 3.1.2. Lotka–Volterra with Seasonal Forcing

As another benchmark we considered an extension of a classical predator-prey model, namely, a Lotka–Volterra model including seasonal forcing of the predation rate, using two additional parameters that control the amplitude (*ε*) and phase (*ω*) of the forcing:

$$x_{1}^{\prime }(t)=\alpha x_{1}(t)-\beta (1+\varepsilon \sin(2\pi (t/T+\omega )))x_{1}(t)x_{2}(t),\;x_{2}^{\prime }(t)=\delta (1+\varepsilon \sin(2\pi (t/T+\omega )))x_{1}(t)x_{2}(t)-\gamma x_{2}(t).$$

The nonlinear parameters are *∊* and *ω*. We considered the dynamics within the time interval *t* ∈ [0, 25]. The parameter setup is given by

θ={α,β,γ,δ,ε,ω}={2/3,4/3,1.0,1.0,0.2,0.5},

and initial values are {*x*_1_(0), *x*_2_(0)} = {0.9, 0.9}. Four experimental scenarios were studied, corresponding to sample sizes of 100 and 200, and SNRs of 10% and 20%. The prior information values were {0.05, 0.1, 0.2}, corresponding to high, medium, and low quality, respectively. The size of the Monte-Carlo study was 500 simulations.

#### 3.1.3. GMA System

The GMA system we analyzed consists of three differential equations in three variables ([18]; pp. 84–85). They are

$$x_{1}^{\prime }(t)=\gamma_{11}x_{2}^{f_{121}}(t)x_{3}^{f_{131}}(t)-\gamma_{12}\;\;x_{1}^{f_{112}}x_{2}^{f_{122}}-\gamma_{13}x_{1}^{f_{113}}x_{3}^{f_{133}},\;x_{2}^{\prime }(t)=\gamma_{12}x_{1}^{f_{112}}x_{2}^{f_{122}}-\gamma_{22}x_{2}^{f_{222}},\;x_{3}^{\prime }(t)=\gamma_{13}x_{1}^{f_{113}}x_{3}^{f_{133}}-\gamma_{32}x_{3}^{f_{332}}.$$

Here the linear parameters are the rate constants *γ*, while the nonlinear ones are the indexed kinetic orders *f*. Note that the parameters *f* are allowed to become negative and their sign might or might not be known. We considered the dynamics of the system within the time interval [0, 4]. The parameter setup is the one presented in Voit [18]; namely,

$$\theta =\{\gamma_{11},f_{121},f_{131},\gamma_{12},f_{112},f_{122},\gamma_{13},f_{113},\;\;f_{133},\gamma_{22},f_{222},\gamma_{32},f_{332}\}\;\;=\{0.4,-1.0,-1.0,3.0,0.5,\;\;-0.1,2.0,0.75,-0.2,1.5,0.5,5.0,0.5\},$$

and initial values are {*x*_1_(0), *x*_2_(0), *x*_3_(0)} = {0.5, 0.5, 1.0}. Four experimental scenarios were studied: sample sizes of 100 and 200, with SNRs of 10% and 20%. The prior information values were {0.1, 0.3, 0.5}, corresponding to high, medium, and low quality, respectively. The size of the Monte-Carlo study was 500 simulations. Parameter estimation for GMA systems is considered to be a challenging numerical task [18].

#### 3.1.4. FitzHugh–Nagumo System

The FitzHugh–Nagumo (FHN) system [51–53] models spiked action potentials in neuron transmission. It is given by

(10) $$x_{1}^{\prime }(t)=c\left(x_{1}(t)-\frac{x_{1}^{3}(t)}{3}+x_{2}(t)\right),\;x_{2}^{\prime }(t)=-\left(\frac{1}{c}\right)\left(x_{1}(t)-a+bx_{2}(t)\right).$$

This system with two state variables was proposed as a simplification of a more complicated model presented in Hodgkin and Huxley’s study [54] for studying and simulating nerve function in giant squid axons. The FHN model is used in neurophysiology as an approximation of the observed action potential.

The system (10) is linear in parameters *a*, and *b*, but nonlinear in *c*. We considered two sample sizes, *n* = 20 and *n* = 40, and two SNRs of 10% and 20%. The parameters were set to {*a*, *b*, *c*} = {0.2, 0.2, 3}. The initial values were {*x*_1_(0), *x*_2_(0)} = {−1.0, 1.0}. The true solutions were obtained over the time interval [0, 20]. The prior information used here was {0.5, 1.0, 3.0}, corresponding to high, medium, and low quality, respectively (the initial guesses for parameters were assured to be positive). The size of the Monte-Carlo study was 500 simulations. Many researchers studied the problem of parameter estimation for the FHN model. In particular, Ramsay et al. [55], Campbell and Steele [56], and Ramsay and Hooker [11] pointed out several difficulties in estimating the parameters for this ODE system.

### 3.2. Results of the Monte-Carlo Analysis

Our findings are presented through charts and tables. The primary summaries are Tables 1 and 2, where we report the ratios of the mean square errors (square errors averaged over Monte-Carlo simulations) for estimates of linear parameters (for nonlinear parameters, see the discussion at the end of this section). Several conclusions can be gleaned from the tables.

- Given high-quality prior information, the accuracy of NLS and SLS is comparable, and neither is superior throughout the variety of experimental setups (at least some of the differences that one sees from the raw numbers in the tables are plausibly attributable to the Monte-Carlo simulation error and as such appear to be insignificant).
- When the quality of prior information degrades to medium or low, the performance of SLS becomes in most cases significantly better than that of NLS (with an extent depending on the specific experimental setup).
- For a fixed noise level, as the sample size increases, the advantage of SLS becomes more pronounced.
- For a fixed sample size, as the noise level increases, the SLS is still better than NLS, but to a lesser extent.

**FIGURE 1:**
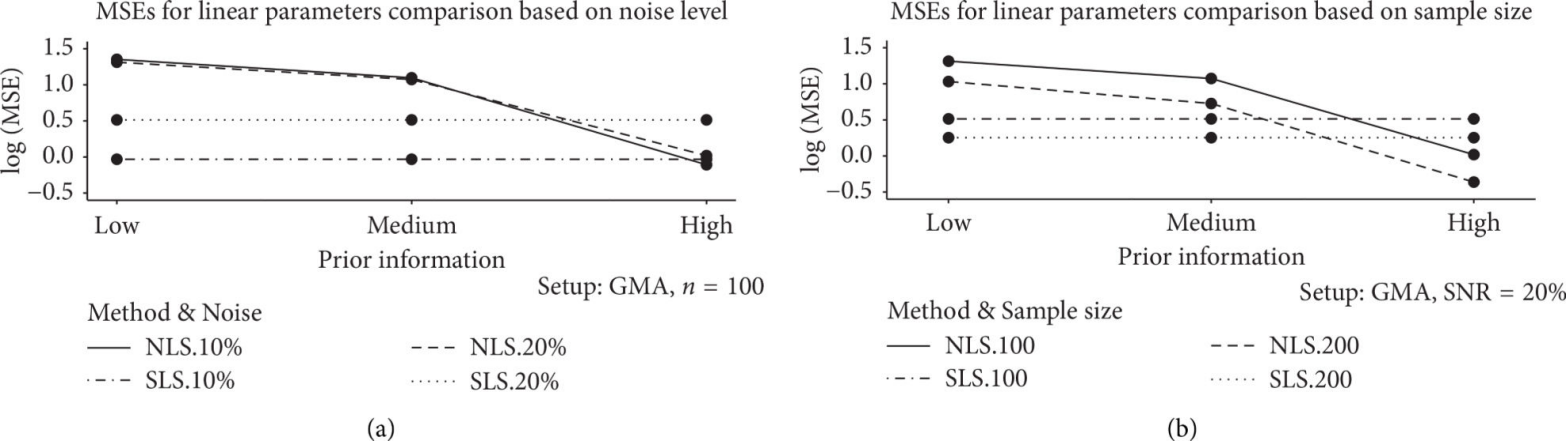
The plot gives MSEs on a log scale (computed as averages over Monte-Carlo simulation runs) for linear parameters plotted against the quality of prior information. In panel (a), the comparison is on the basis of the noise level. The graph indicates that the performance of NLS worsens with decreasing quality of prior information. On the other hand, the performance of SLS is not affected by the quality of prior information, in agreement with the experimental design. Except for the rare case of high-quality prior information, where NLS is better, SLS clearly outperforms NLS. In panel (b), the comparison is based on the sample size. The overall pattern is similar to that in panel (a).

These results can also be visualized through a combination of simple statistical charts. Thus, Figure 1 displays the line graphs that compare MSEs of the two methods under several experimental setups. Whereas the numbers in Tables 1 and 2 are ratios of MSEs, the figures here present absolute MSE values. From the graphs, an advantage of SLS over NLS is apparent for less than ideal prior information. Note that, in this specific setting, SLS performed worse than NLS for high-quality prior information. A plausible explanation may be the following: while, under our experimental setup, the amount of information used by SLS via (3) is fixed throughout simulations, NLS can in principle receive arbitrarily precise initial guesses on linear parameters. One may therefore envision a threshold, where using the latter kind of information outweighs the benefits of using the structural relationship (3). However, a precise quantification of the phenomenon is hardly possible beyond an observation that it appears to manifest itself in scenarios with excellent knowledge on likely parameter values. In reality, such ideal prior information is rare.

Panel (a) of Figure 1 further suggests that in the specific scenarios we report, SLS improves when the noise level decreases, which is different from NLS in the same figure.

**FIGURE 2:**
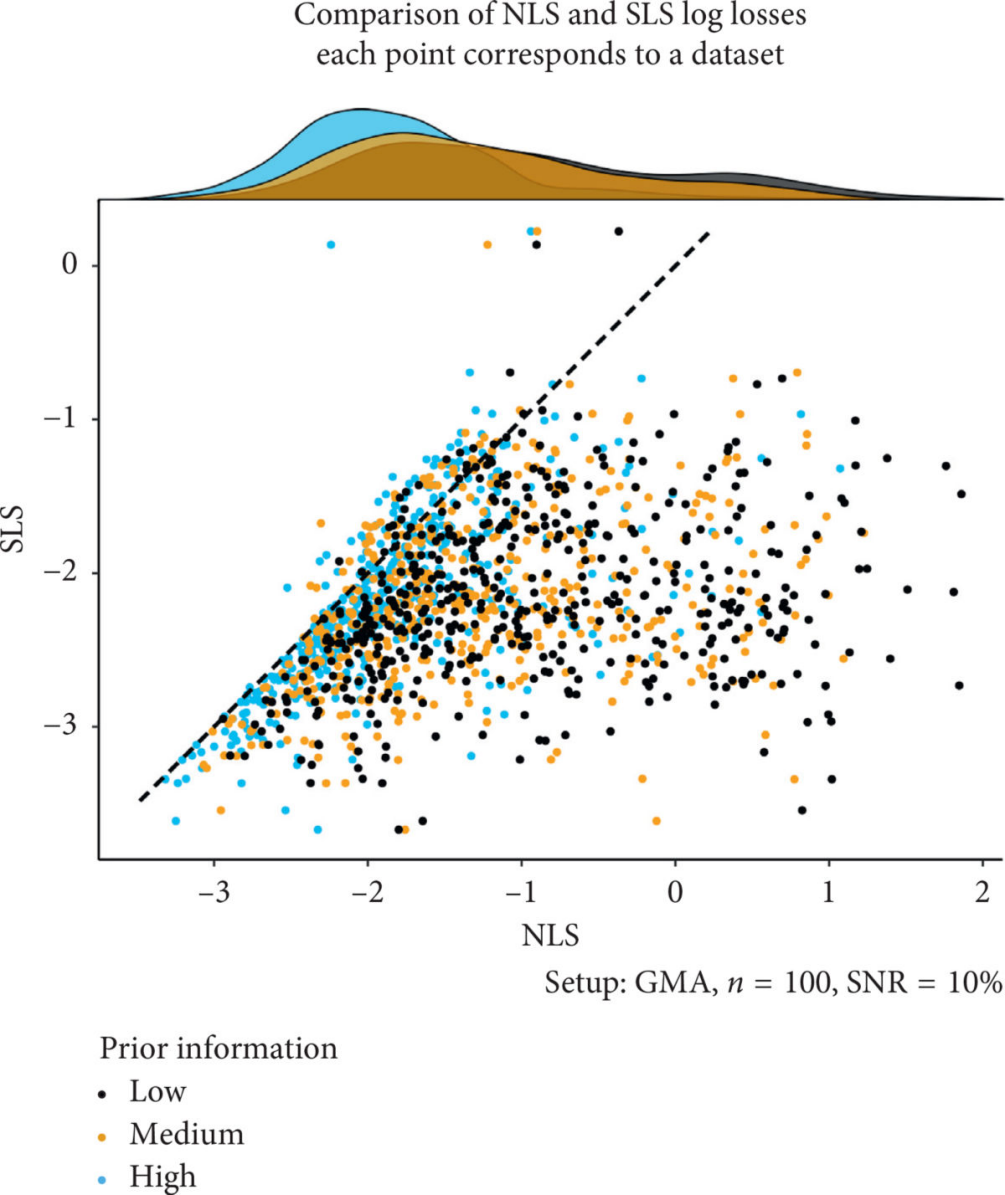
The plot visualizes the performance (on a log scale) of NLS and SLS according to criteria (5) and (7), which are evaluated at the optimal parameter estimates. Points in the scatterplot are colored according to the quality of prior information used to compute the NLS estimates. The 45° diagonal line passing through the origin has been added for reference and intuitive assessment. The scatterplot is supplemented with marginal density estimates using the same color coding. The density estimates indicate that, as the quality of prior information degrades, the quality of NLS results suffers, which manifests in longer right tails of the densities. By definition, performance of SLS is not affected by the quality of prior information on linear parameters. For high-quality prior information, clustering of losses in the scatterplot close to the reference line suggests that the overall performance of both NLS and SLS is comparable. As the quality of prior information decreases, the point clouds spread to the right, indicating that SLS starts to perform noticeably better than NLS. Furthermore, unlike Tables 1 and 2, the scatterplot and the range frame (see [57]; pp. 130–132) convey an impression of the variability in the estimation results over multiple datasets: NLS is visually more variable than SLS.

Figure 2 is a scatterplot of NLS and SLS losses (5) and (7) (on a log scale) evaluated at optimal parameter estimates. The figure highlights in yet another manner the importance of prior information for NLS: it is evident that the performance of the latter is strongly affected by the quality of initial parameter guesses. Again, NLS and SLS perform similarly when the prior information is of high quality. However, when the quality of prior information is less than ideal, as it is in most applications, NLS becomes substantially worse than SLS. The scatterplot also gives a quick impression of the variability of estimation results.

**FIGURE 3:**
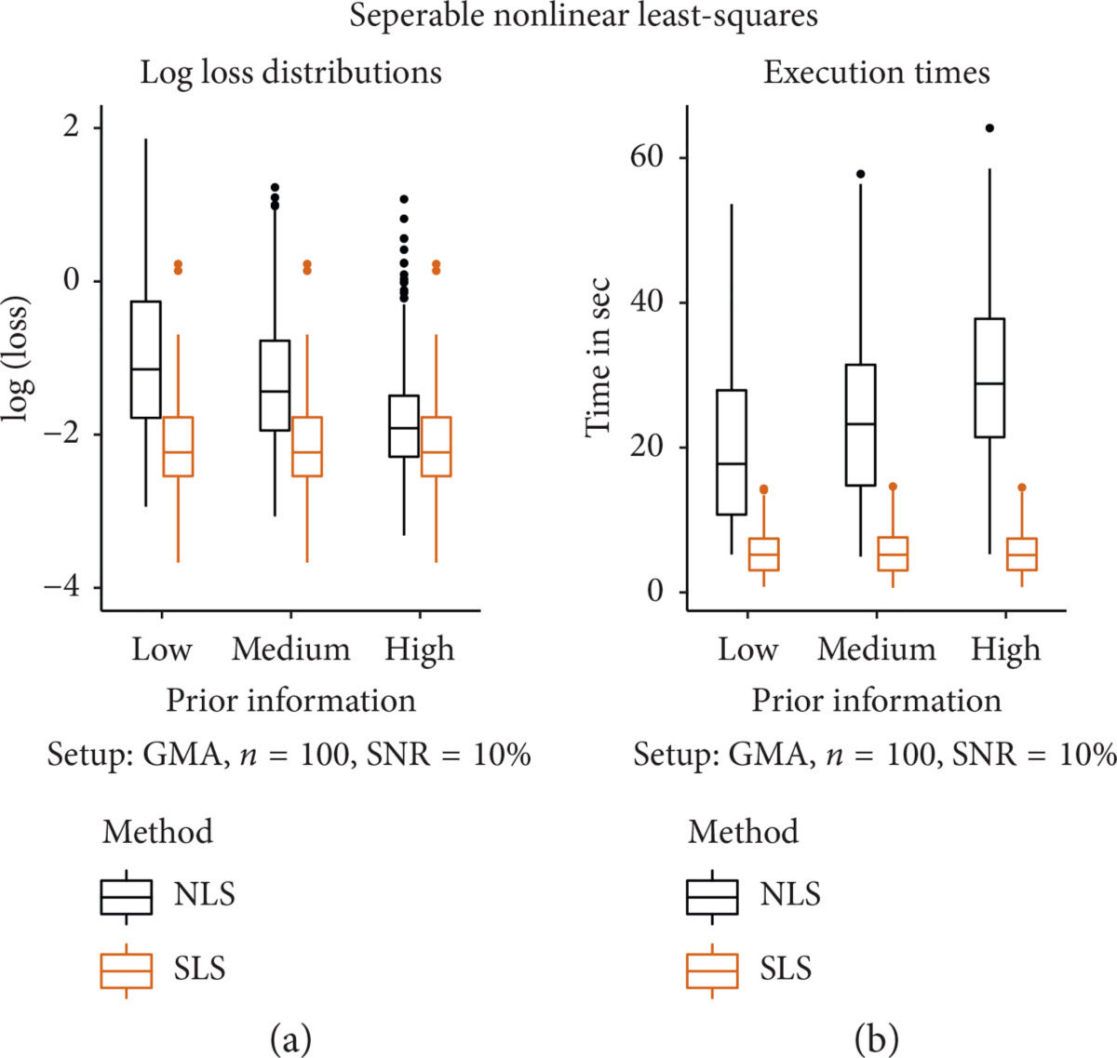
The plot presents a comparison of NLS and SLS. In panel (a) boxplots of the losses (5) and (7) (on a log scale) evaluated at the optimal parameter estimates are displayed. For high-quality prior information, the NLS and SLS loss distributions are close. As the quality of prior information degrades, NLS losses start to assume higher values compared to SLS, and their variability increases, as evidenced by the elongation of boxplots. In panel (b) the computation times are compared. The NLS computation times tend to be longer than those of SLS and increase as the quality of prior information increases. In both panels, the performance of SLS does not vary with the quality of prior information, in concordance with the experimental design.

The conclusions that we drew from Figure 2 are confirmed by the panel (a) of Figure 3, which presents boxplots of NLS and SLS losses (on a log scale) measured according to criteria (5) and (7). The pattern is clear: SLS performs better than NLS, and the inferiority for NLS becomes more dramatic with degrading prior information.

Panel (b) of Figure 3 summarizes computation times. SLS is in general much faster. The execution time of NLS is affected by the quality of prior information and, interestingly, increases with this quality. The results for all other models (and noise levels) were similar and are therefore omitted.

Finally, Tables 3 and 4 provide information regarding the nonlinear parameters. In the case of NLS, one can observe how prior knowledge regarding linear parameters propagates into the estimation accuracy for nonlinear parameters. In particular, for less than ideal prior information on the linear parameters, SLS holds a pronounced edge over NLS, even in the case of nonlinear parameters.

## 4. Outlook

Data fitting in complex dynamical systems remains a challenging problem that cannot be treated in a cavalier fashion, even if one takes advantage of separability. For instance, in order to uncover the patterns in Section 3 of this work, we had to carefully design the experimental study, because otherwise simulations might not have converged or might have converged to poor solutions. This was true for both NLS and SLS, but whenever they were observed, convergence issues were much more severe for NLS; they were especially sensitive in the case of the FitzHugh–Nagumo system. This result highlights the crucial role of *prior information* regarding the parameters or, expressed differently, the quality of the initial parameter guesses used for the optimization. We focused here primarily on the effects of the prior information regarding the linear parameters. However, it also became clear that prior information on the nonlinear parameters has an equally crucial role for optimization purposes, and this was true for both NLS and SLS (data not shown).

As a result of our exploratory work, we envision the following promising research directions for the future.

### 4.1. Numerical Implementation of SLS for Dynamic Systems

All computations in our analysis were done in *R* using the *simode* package of Yaari and Dattner [25]. However, the idea of using separability properties of ODEs is independent of a particular programming language and can be implemented within other software packages quite as well. Indeed, much work has been done in the context of the variable projection method since it was first introduced by Golub and Pereyra [21]. In the context of nonlinear regression, the variable projection method of Golub and Pereyra [21] is implemented in *R* in the *nls* command; see Venables and Ripley [58] and pp. 218–220 for an example of its application. In addition, we are aware of the *TIMP* package of Mullen and van Stokkum [59]; which implements the variable projection method. Thus, a next step could be to combine the strengths of different packages, e.g., *simode* and *TIMP*, in order to develop advanced software for variable projection in the context of dynamic systems.

### 4.2. Customized Algorithms for Specific Classes of Complex Dynamical Systems

It is well known that the performance of an optimization scheme depends crucially on the underlying mathematical model used for description of the data. Thus, it appears that different classes of dynamic models require specific algorithms tailored to their peculiarities. For instance, parameter estimation for GMA systems has different challenges than those encountered when working with SIR (see Section 3). We expect that there is much to gain from focusing future research on specific classes of models and developing stable algorithms for their parameter estimation.

### 4.3. Theoretical Properties of SLS in the Context of Dynamic Systems

Gugushvili and Klaassen [37] studied the statistical properties of NLS in the general context of smoothing, while Dattner and Klaassen [20] specifically addressed ODE systems that are linear in (functions of) the parameters. One might expect that some assumptions used in Gugushvili and Klaassen [37] can be relaxed when the problem is closer to the one considered in Dattner and Klaassen [20].

### 4.4. Extensions to Partially Observed, High-Dimensional, and Misspecified Dynamic Systems

Recent work dealing with inference in high-dimensional ODE models suggests that exploiting linearity in parameters is crucial for developing a successful estimation methodology (see e.g., [24, 39]). More generally, it would be interesting to use the variable projection method to study cases of partially observed, high-dimensional, and possibly misspecified dynamic systems. This work might additionally require the use of high-dimensional regularization techniques (e.g., [39]) for balancing data and model, and specifically take into account a potential model misspecification (see [55]).

## 5. Conclusions

In this work, we designed an extensive simulation study to explore the relative statistical and computational performance of two optimization schemes for inference in dynamic systems: the typical nonlinear least-squares (NLS) method and a novel, separable nonlinear least-squares (SLS) approach. As benchmarks, we considered several widely used ODE models arising in a variety of biological fields. We measured statistical performance of the two methods by the mean square error (MSE) of the estimates. As another performance criterion, we employed the loss function values at the optimal parameter estimates. Computational performance of the methods was also compared by the execution times required to complete each optimization.

Our overall recommendation is the following: whenever a complex dynamic system contains an appreciable number of linear parameters, estimation of its parameters should be addressed with the separable nonlinear least-squares method, rather than the more commonly used, generic nonlinear least-squares method. The general pattern emerging from our study is that SLS performs at least as well as, and frequently better than, NLS, especially if the prior information regarding the system is not ideal, which is typically the case in practice. This statement was found to be uniformly true over all models tested.

## Figures and Tables

**Table 1:** MSE ratios for linear parameters (small samples).

Prior		Low noise				High noise		
	SIR	LV	GMA	FHN	SIR	LV	GMA	FHN

Low	8.3	5.8	4.0	2.3	2.8	2.2	2.2	1.3
Medium	3.9	1.8	3.1	1.7	1.4	1.2	1.8	1.2
High	0.9	1.3	0.9	2.0	0.4	1.0	0.6	1.1

The MSE ratios (computed by averaging square errors over Monte-Carlo simulation runs) of NLS and SLS for estimating the linear parameters in various benchmark models and under different experimental setups are displayed (see Section 3.1 for detailed specifications). To identify model names, self-explanatory abbreviations are used. The values in the table are rounded off to one significant digit. The sample size is *n* = 100 for the GMA and Lotka–Volterra models, *n* = 20 for the FitzHugh–Nagumo system, and *n* = 18 for SIR model. The noise levels are 10% and 20%. Values larger than 1 in the table correspond to the cases where SLS performs better than NLS. Note the decreasing pattern in the columns, reflecting the effect of the quality of prior information on the performance of NLS.

**Table 2:** MSE ratios for linear parameters (large samples).

Prior		Low noise				High noise		
	SIR	LV	GMA	FHN	SIR	LV	GMA	FHN

Low	12.0	9.6	3.9	3.9	3.8	3.2	2.2	2.2
Medium	4.1	4.3	2.8	1.9	1.6	1.6	1.6	1.3
High	1.0	2.2	0.7	2.3	0.7	1.2	0.5	1.5

The sample size is *n* = 200 for the GMA and Lotka–Volterra models; *n* = 40 for the FitzHugh–Nagumo system; and *n* = 36 for the SIR model. The noise levels are 10% and 20%. For an interpretation of the results, see Table 1. There is an increased advantage of SLS over NLS in comparison to Table 1.

**Table 3:** MSE ratios for nonlinear parameters (small samples).

Prior		Low noise				High noise		
	SIR	LV	GMA	FHN	SIR	LV	GMA	FHN

Low	23.0	1.7	1.1	1.1	6.6	1.1	1.0	1.0
Medium	8.7	1.2	1.0	1.0	2.6	0.9	1.0	1.0
High	1.0	1.0	0.9	1.0	0.4	0.9	0.9	1.0

The table displays the MSE ratios (computed through squared errors averaged over Monte-Carlo simulations) of NLS and SLS for estimating the nonlinear parameters. The experimental setup is as in Table 1. Values larger than 1 in the table correspond to the cases where SLS performs better than NLS. Since the prior information regarding nonlinear parameters stays invariant (see Section 3.1 for details), the table in particular shows the effects that the quality of initial guesses for linear parameters has on the estimation accuracy of NLS in the case of nonlinear ones. The results suggest that, in some settings, vague prior knowledge regarding linear parameters may have an adversary effect on the accuracy of NLS with respect to the nonlinear parameters.

**Table 4:** MSE ratios for nonlinear parameters (large samples).

Prior		Low noise				High noise		
	SIR	LV	GMA	FHN	SIR	LV	GMA	FHN

Low	29.0	4.1	1.1	1.6	6.5	1.3	1.0	1.3
Medium	8.2	2.1	0.9	1.0	2.2	1.1	0.9	1.0
High	0.9	1.2	0.8	1.0	0.7	0.9	0.8	1.0

The setup is as in Table 2. For an interpretation of the results, see Table 3.
